# Malignancies After Renal Transplantation: Frequency, Etiology, and Prognosis—A Single Center Experience

**DOI:** 10.3390/jcm14165858

**Published:** 2025-08-19

**Authors:** Fatih Atalah, Aydın Acarbay, Akgün Karakök, Mehmet Beşiroğlu, Fatih Kuş, Huzeyfe Arıcı, Ahmet Burak Dirim, Vafa Suleymanova, Aydın Türkmen, Halil Yazıcı

**Affiliations:** 1 Division of Medical Oncology, Department of Internal Medicine, Faculty of Medicine, Istanbul Medeniyet University, 34720 Istanbul, Türkiye; aydinacarbay@gmail.com (A.A.); akgunkarakok@gmail.com (A.K.); mbesiroglu@yahoo.com.tr (M.B.); 2 Department of Medical Oncology, Ankara Atatürk Sanatoryum Eğitim ve Araştırma Hastanesi, 06290 Ankara, Türkiye; fatihkush@hotmail.com; 3 Department of Internal Medicine, Faculty of Medicine, Istanbul University, 34452 Istanbul, Türkiye; huzeyfearicii@gmail.com (H.A.); ahmetburakdirim@gmail.com (A.B.D.); vafasuleymanova@gmail.com (V.S.); turkmenaydin@yahoo.com (A.T.); halildrr@yahoo.com (H.Y.)

**Keywords:** malignancy, mortality, post-transplant malignancy, prognosis, renal transplantation

## Abstract

**Introduction and Aim**: Renal transplant recipients face significant long-term graft and patient loss due to post-transplant malignancies. This study aimed to characterize post-transplant malignancies, determine mortality risk factors, and evaluate patient outcomes. **Materials and Methods**: This retrospective study included 2052 kidney transplant recipients who underwent transplantation between 1976 and 2019 at our institution, other national centers, or international facilities, and who had at least six months of follow-up. Regardless of the transplant center, all patients were followed exclusively at our nephrology department for post-transplant care. A comprehensive review of patient files was conducted, encompassing demographic data, malignancy type and treatment, mortality rates, tissue compatibility assessments, viral serology results, immunosuppression protocols, acute rejection history, and pre-transplant malignancies. The relationships between these variables and mortality were examined. **Results**: A total of 167 malignant events were observed in 163 patients out of 2052 renal transplant patients (7.9%). The female patients comprised 34.4% (*n* = 56) of the participants. Ages at transplantation and malignancy diagnosis had medians of 40.0 (13–72) and 50.0 (23–78) years, respectively. The leading malignancy was skin cancer at 30.0%, with Kaposi sarcoma at 11.3% and post-transplant lymphoproliferative disease at 10.6% following. Of the patients followed up, 58.9% (93 patients) had mortality. In univariate analysis, older age at transplant, older age at malignancy diagnosis, and male sex were associated with mortality; however, no independent predictors were identified in the multivariate model (all *p* > 0.05), likely due to sample size limitations and inter-variable collinearity. Mortality showed statistically significant associations (*p* < 0.05) with increased age at transplantation, increased age at malignancy diagnosis, and male gender. **Conclusions**: Post-transplant malignancies significantly compromise both graft longevity and patient survival. Particularly aggressive skin cancers demand heightened clinical vigilance. Early detection through regular dermatological screening, patient education, and timely biopsies must become integral to long-term transplant care protocols.

## 1. Introduction

Impaired kidney structure or function defines chronic kidney disease (CKD), a progressive condition with symptoms varying by cause and stage [1]. Progressive CKD is influenced by primary disease, genetics, and demographics, but its progression remains consistent. End-stage renal disease (ESRD), the final stage of CKD, is marked by severely impaired kidney function. Renal replacement therapy (RRT) is necessary for the survival of patients who have reached this stage. The RRTs comprise renal transplantation, hemodialysis, and peritoneal dialysis [2]. In treating ESRD, renal transplantation is superior in improving life quality and prolonging survival for patients [3].

In 2023, worldwide solid organ transplantations reached 172,409, as per Global Observatory on Donations and Transplantation (GODT) data. From this group, 111,135 involved renal transplants [4]. Post-transplant graft survival has improved dramatically thanks to better surgical methods and immunosuppressant drugs, achieving a 90% one-year survival rate [3,5,6]. Data from the Scientific Registry of Transplant Patients (SRTR) in 2023 shows renal transplant recipients have a 5-year survival rate exceeding 90% [6]. For post-renal transplant, though, a number of medical and surgical complications are possible, such as heart disease, infections, and the development of malignancies. The survival of both the graft and patient is negatively impacted by these complications.

Long-term data on post-transplant malignancies in dialysis and renal transplant patients are most comprehensively provided by the Australia and New Zealand Dialysis and Transplant (ANZDATA) registry. Post-transplant malignancy rates rise annually, reaching 20% by year 10 [5]. In the coming years, post-transplant malignancy is likely to become the leading cause of death among transplant recipients because of increased lifespans, an aging patient population, and long-term immunosuppressant use.

Post-transplant malignancy risk is 2–4 times greater than in the general population, according to numerous studies [7]. Transplant patients have a lower incidence of common malignancies like lung, breast, prostate, and colon adenocarcinoma compared to the general population [8]. On the other hand, the risk of rare malignancies associated with oncogenic viruses, such as non-Hodgkin lymphoma and Kaposi sarcoma, is close to 100-fold [3,9,10]. Advanced-stage detection is common for post-transplant malignancies, which tend to be aggressive.

Post-transplant malignancy risks encompass general factors (like smoking, sun exposure, and malignancy history) and transplant-specific factors. The transplant-specific factors were immunosuppression level, immunosuppressive agent, oncogenic viruses, and renal disease risk factors [11,12]. In some cases, post-transplant malignancy may also be of donor origin. Moreover, the length of pre-transplant dialysis also increases the risk [13].

Renal transplant recipients diagnosed with pre- or post-transplant malignancies at our clinic between 1976 and 2019 were studied for clinical characteristics, malignancy types, treatments, and survival outcomes. To guide future transplant oncology management, this study identified demographic, immunological, and therapeutic factors linked to mortality.

## 2. Materials and Methods

### 2.1. Study Design and Population

This retrospective study included 2052 adult kidney transplant recipients who underwent transplantation between 1976 and 2019 and were followed for at least 6 months post-transplant. Patients with a follow-up duration of <6 months and those lost to follow-up for reasons other than graft loss or death after malignancy diagnosis were excluded. The study population comprised 163 recipients diagnosed with malignancy either before or after transplantation. Supplemental Figure S1 shows the study cohort selection and follow-up flow chart.

Electronic medical records and patient charts provided the data. Renal transplant recipients diagnosed with any malignancy, before or after transplantation, met the inclusion criteria. The study excluded patients with less than 6 months of follow-up and any patients lost to follow-up unless due to graft loss or death following a malignancy diagnosis. This study followed the Declaration of Helsinki, and the University Faculty of Medicine Local Ethics Committee approved the study (Decision protocol no.: 3354094, Date: 11 June 2025). All participants provided informed consent.

### 2.2. Data Collection

The demographic data collected included age at transplantation, sex, duration of renal replacement therapy, type and duration of pre-transplant dialysis, and smoking status. The estimated glomerular filtration rate (eGFR) of patients was documented as milliliters [mL] per minute [min] [14]. Transplant variables considered included donor type (living or deceased), HLA compatibility (high [>3/6] or low [≤3/6]), induction therapy (Antithymocyte globulin/Antilymphocyte globulin [ATG/ALG] or anti-Interleukin 2 [IL2] agents), maintenance immunosuppression (steroids, calcineurin inhibitors [CNI], antiproliferative agents, and Mammalian Target of Rapamycin [mTOR] inhibitors), history and number of acute rejection episodes, and graft loss. Induction regimen data were available for 108 patients (ATG/ALG: 47; anti-IL-2: 4; no induction/no record: 57). Percentages were calculated based on the number of patients with available data. HLA compatibility data were available for 116 patients. Data were missing for 47 patients due to incomplete historical records, and these individuals were excluded from HLA compatibility analyses. Data on Epstein–Barr virus (EBV), Cytomegalovirus (CMV), hepatitis B (HBV), and hepatitis C (HCV) serology and BK virus (BKV)/CMV infection post-transplant were collected.

### 2.3. Malignancy Assessment

Malignancies were classified into pre-transplant (diagnosed before the index kidney transplant) or post-transplant (diagnosed after the index transplant). Overlap indicates patients who had both pre- and post-transplant malignancies. The post-transplant malignancies were divided into these groups: non-Kaposi sarcoma skin tumors, Kaposi sarcoma, post-transplant lymphoproliferative disorder (PTLD), urinary system tumors, other solid tumors, hematological malignancies, and premalignant lesions. Histological examination confirmed malignancy in all cases. PTLD cases showed specific instances of organ dysfunction and CNS involvement.

### 2.4. Treatment Modalities

Malignancy treatments included surgery alone, surgery combined with chemotherapy (CT) and/or radiotherapy (RT), CT alone, RT alone, reduced immunosuppression, surgery plus radioactive iodine, or observation without active treatment. Changes to immunosuppression after a malignancy diagnosis (like adding mTOR inhibitors) were recorded.

### 2.5. Outcomes

The primary outcome was overall mortality during the follow-up period. Relationships between malignancy type, graft survival, and overall survival were also secondary outcomes of the study. In this study, resumption of renal replacement therapy was considered as graft loss. At the last follow-up, we collected data on mortality, malignancy treatment outcomes, and graft function. The electronic data system provided the mortality and mortality dates. The final follow-up date for non-exitus patients was set as February 2025.

### 2.6. Statistical Analysis

Statistical analyses of the study data were performed with the Statistical Package for the Social Sciences program (SPSS) version 22.0. Mean, standard deviation, median, minimum, maximum, frequency, and ratio were presented as descriptive statistics for the data. Variable distribution was assessed using the Kolmogorov–Smirnov test. Inferential statistics determined relationships and differences between groups. The Shapiro–Wilk test determined numerical data normality for test selection, which also considered test assumptions. T-tests compared two independent groups of numerical data if they were normally distributed; ANOVA compared more than two. When the data were not normally distributed, the Wilcoxon rank-sum test (two groups) or Kruskal–Wallis test (three or more groups) was employed. Chi-square tests were applied to categorical data when cell counts exceeded five; otherwise, Fisher’s exact test was used. Results were considered statistically significant when the *p*-value was below 0.05.

## 3. Results

### 3.1. Baseline Demographic and Clinical Characteristics

This study comprised 163 individuals who had undergone renal transplantation. The median follow-up duration was 157 months (7–531). The median age at transplantation was 40 years (13–72), and 65.5% of the individuals were male. Pre-transplant dialysis was required in 87.7% of the patients, with hemodialysis being the most common modality (76.5%). Of all transplantations, 67.5% of organs came from living donors and 32.5% from deceased donors. Table 1 presents the demographic characteristics of transplant patients.

Patients underwent a median of 84 months (13–156) of dialysis before transplantation, and their median eGFR at diagnosis was 59.0 mL/min (8.0–117.0). Low HLA histocompatibility was present in 79.3% of patients. In 7.7% of patients, acute rejection was observed, primarily during the first year following transplantation.

### 3.2. Malignancy Profile

Among the 163 recipients with any malignancy, 11 patients (6.7%) had a pre-transplant malignancy and 160 patients (98.2%) had a post-transplant malignancy (Table 2). Three patients (1.8%) were diagnosed with both a pre- and a post-transplant malignancy—two were recurrences (multiple myeloma and basal cell carcinoma), and one was a new lymphoma (in a renal cell carcinoma patient). When overlap is taken into account, this corresponds to 8 patients with pre-transplant malignancy only, 157 patients with post-transplant malignancy only, and 3 patients in both groups. Pre-transplant malignancies consisted of non-melanoma skin cancer, breast cancer, colon cancer, renal cell carcinoma (RCC), bladder cancer, intracranial tumors, and various other cancers.

**Figure 1:**
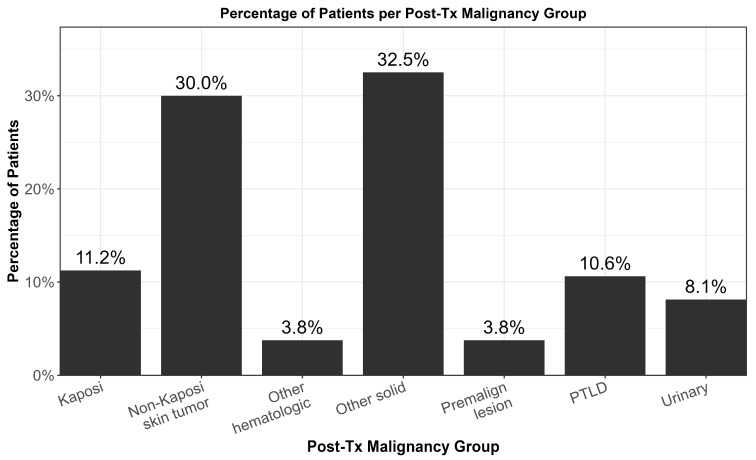
Distribution of post-transplant malignancy types among included recipients.

Post-transplant malignancies occurred in 95.8% of patients; the most prevalent were non-Kaposi sarcoma skin tumors (30%), followed by Kaposi sarcoma (11.3%), PTLD (10.6%), urinary tract tumors (8.1%), other solid tumors (32.5%), and hematological malignancies (3.8%). Premalignant lesions were found in 3.8% of participants. At diagnosis, 52.9% of PTLD cases showed organ dysfunction, while 23.5% involved the central nervous system. Post-transplant malignancy types diagnosed in transplant recipients are illustrated by their distribution in Figure 1.

### 3.3. Comparison of Malignancy Types

Compared to patients with urinary (66.2 ± 12.5 years) or other skin tumors (62.0 ± 8.8 years), Kaposi sarcoma patients were diagnosed at a younger age (mean: 59.3 ± 12.8 years). Male predominance was observed in Kaposi sarcoma and non-Kaposi skin tumors regarding sex distribution, varying by malignancy type (Table 3).

Post hoc comparisons of mean differences were conducted across tumor and lesion groups, referencing urinary tumors. Several comparisons showed statistically significant differences following Bonferroni correction. The most significant mean difference was observed between premalignant lesions and urinary tumors (mean difference = 25.38, 95% CI: 9.93–40.83, *p* < 0.0001, *p* < 0.0001 after Bonferroni correction). Similarly, Kaposi sarcoma (mean difference = 18.85, 95% CI: 7.46–30.24, *p* < 0.0001, Bonferroni *p* < 0.0001), PTLD (mean difference = 18.49, 95% CI: 6.96–30.03, *p* = 0.0001, Bonferroni *p* = 0.0021), and other solid tumors (mean difference = 14.89, 95% CI: 5.19–24.60, *p* = 0.0002, Bonferroni *p* = 0.0042) were also significantly different from the urinary tumor group.

Induction regimens differed considerably depending on the type of malignancy. The use of ATG or ALG induction therapy was notably higher in PTLD patients (52.9%) compared to Kaposi sarcoma patients (16.7%), as detailed in Table 3.

### 3.4. Mortality Analysis

Table 4 summarizes participant statistics categorized by mortality, revealing mortality in 57.0% (*n* = 93) of participants. Compared to survivors, non-survivors were significantly older at both transplantation (43.7 vs. 37.3 years, *p* < 0.001) and malignancy diagnosis (52.6 vs. 45.6 years, *p* < 0.001). In addition, the time spent on renal replacement therapy before transplantation was considerably shorter for non-survivors (36 vs. 84 months, *p* = 0.018).

A significant association was found between EBV seropositivity and mortality (32.9% vs. 16.1%, *p* = 0.026), with no significant difference observed for CMV, HBV, or HCV. Non-survivors showed significantly higher rates of cyclosporine A (77.4% vs. 58.6%, *p* = 0.017) and azathioprine (54.8% vs. 38.6%, *p* = 0.006) use than survivors among various immunosuppressive treatments. Non-survivors exhibited a statistically significantly greater prevalence of Kaposi sarcoma (33.0% vs. 25.4%) and PTLD (12.1% vs. 9.5%) than survivors.

**Figure 2:**
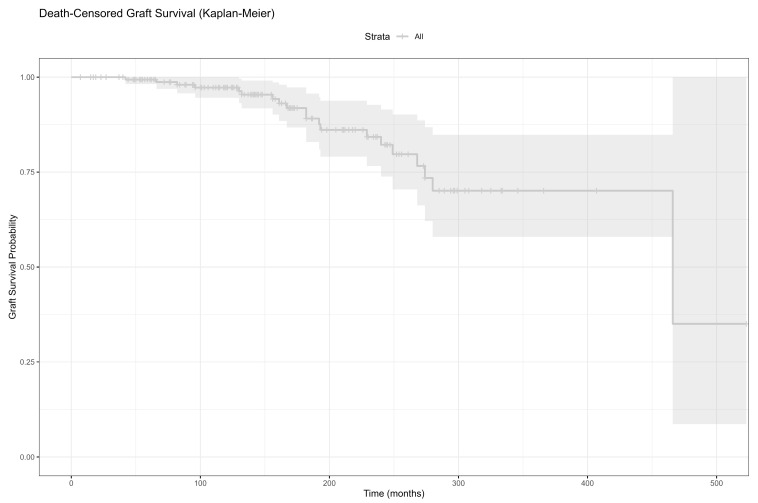
Death-censored graft survival with number at risk at 0-, 60-, 120-, and 180-months post-transplant.

As shown in Figure 2, the death-censored graft survival progressively declined over time, with the numbers at risk detailed for 0, 60, 120, and 180 months post-transplantation.

### 3.5. HLA Compatibility and Malignancy

HLA compatibility in pre- and post-transplant malignancy groups is evaluated in Table 5. There was no significant association between HLA matching and malignancy type before transplantation (*p* > 0.999).

### 3.6. Independent Risk Factors of Mortality in Post-Transplant Malignancy Patients

Independent predictors of mortality among transplant patients diagnosed with post-transplant malignancy were identified using multivariate logistic regression analysis (Table 6). Statistical analysis revealed no significant link between mortality and age at the time of transplantation, EBV seropositivity, use of CNIs, or Antimetabolite in maintenance treatment. The age at the time of transplantation did not have a notable impact on the death risk (OR = 1.026). Age at the time of transplantation did not significantly influence the risk of mortality (OR = 1.026; 95% CI: 0.910–1.156; *p* = 0.678). Likewise, mortality was not significantly associated with EBV seropositivity (OR = 0.260; 95% CI: 0.013–5.356; *p* = 0.383), although the wide CI suggests high variability and uncertainty. Analysis of immunosuppressive treatments indicated a potential mortality benefit with CNIs (OR = 0.132; 95% CI: 0.011–1.591; *p* = 0.111), but this did not reach statistical significance. In univariate analysis, older age at transplant, older age at malignancy diagnosis, and male sex were associated with mortality; however, no independent predictors were identified in the multivariate model (all *p* > 0.05), likely due to sample size limitations and inter-variable collinearity.

## 4. Discussion

Renal transplantation is the top choice for treating ESRD, owing to its superior long-term outcomes [2]. Despite improvements in early patient and graft survival due to new immunosuppressants and surgical techniques, long-term issues like cardiovascular disease, infections, and malignancies are rising [11,12]. Renal transplant recipients experience significantly more malignancies than the general population, primarily due to prolonged immunosuppression and weakened immune system monitoring [15]. Contemporary reviews confirm a ~2–3-fold overall cancer risk in kidney recipients, with the excess largely driven by virus-related cancers and non-melanoma skin cancer, reinforcing our cohort’s pattern [16]. Post-transplant malignancy was found in 7.9% (163/2052) of renal transplant recipients in our study, indicating a major long-term complication. The data are consistent with prior studies showing malignancy is the leading cause of death following transplantation, exceeding cardiovascular disease [17].

Malignancy rates in renal transplant recipients are 3–18%, depending on location and patient population, with a 3- to 8-fold increased malignancy risk [18]. Immunosuppressive patients face malignancy risks stemming from their immunosuppressive regimen, immunosuppression level, viral infections, sun exposure, older transplant age, and occasionally, donor-transmitted malignancy [7,18]. Real-world registry-linked data from Thailand (SIR for all cancers 3.85) underscore geographic variation in excess risks—particularly urothelial and lymphoid tumors—mirroring the heterogeneity we observed across subtypes [19]. Our study of 2052 transplant patients (1976–2019) revealed 167 post-transplant malignancies in 163 patients (8.1%).

Older individuals are more prone to post-transplant malignancies [9,13]. A study of 3521 patients from 10 Northern Italian transplant centers revealed 172 malignancies, with a mean age at diagnosis of 49.8 ± 9.5 years [20]. Among 15183 patients in the 2023 ANZDATA registry, 1642 (10.8%) had malignancies, a rate comparable to those 20–30 years older than the norm [5]. In a study from our country, Arican et al. reported a median patient age of 38 years [9]. Patients in our study had a median age of 60 (21–81) years, older than reported in national and Mediterranean publications regarding malignancy diagnosis. The longer study period (1976–2019) and the aging transplant patient population in our study might explain this difference. Additionally, the discrepancies may be attributed to the older study’s use of more potent immunosuppressants, particularly ATG, and a higher incidence of acute rejection.

The current study revealed that 64.5% of post-transplant malignancies occurred in males. A Swedish cohort study evaluating renal, liver, and other organ transplant recipients revealed that 61% of transplant patients and 63.9% of malignancy patients were male. Another study reported that 66.6% of post-transplant malignancy cases were male [9]. Similar to published data, the present study shows a comparable rate of male patients, potentially explained by higher male transplantation rates in our country.

The frequency of malignancies was highest for non-Kaposi sarcoma skin tumors, followed by Kaposi sarcoma and PTLD in post-transplant individuals [9,10,15,18]. Among transplant recipients, skin cancer is the most frequent malignancy, representing 30–50% of all malignancies [9]. Skin cancer was the most common post-transplantation malignancy in our study, accounting for 30% of cases (*n* = 48). This was followed by Kaposi sarcoma (*n* = 18, 11.3%) and PTLD (*n* = 17, 10.6%). Skin malignancy, Kaposi sarcoma, and PTLD occurred at rates of 25%, 25%, and 22.5%, respectively, in a study in our country [9]. Post-transplant malignancy rates in the current study matched those reported in the literature. Similarly, a recent multicenter analysis reported comparable incidence patterns and emphasized age and immunosuppression intensity as major correlates of risk [13]. Exposure to immunosuppressive therapy and UV radiation raises the chances of oncogenic virus infections, for example, HPV [21]. Likewise, while infrequent in the general population, Kaposi sarcoma shows a higher incidence among transplant recipients, particularly where HHV-8 seropositivity is prevalent [22].

The aggressive nature and high mortality rate of PTLD make it a significant worry. PTLD comprised 10.6% of post-transplant malignancies within our study; over half experienced organ dysfunction, and 23.5% had CNS involvement. Notably, EBV-positive ‘quintessential’ PTLD often responds to lower-intensity approaches, whereas other monomorphic entities behave as de novo lymphomas and require lymphoma-specific chemotherapy [23]. Non-survivors showed significantly increased EBV seropositivity, emphasizing EBV’s critical contribution to PTLD pathogenesis [24]. Recent reviews highlight EBV-driven biology, risk-adapted reduction of immunosuppression, and rituximab-based strategies as first-line approaches, with escalation reserved for refractory disease [25]. Our results agree with earlier studies [26] that stress the significance of post-transplant EBV surveillance, especially for high-risk patients.

A mortality rate of 58.9% was alarmingly observed in our cohort. Older age at transplant and malignancy diagnosis, along with more frequent CsA and AZA use, were associated with non-survival. These results confirm earlier research showing a strong correlation between older age at transplant and higher post-transplant mortality [27]. In addition, first-generation immunosuppressants (CsA and AZA) have shown a stronger link to malignancy development than newer options (tacrolimus and mycophenolate mofetil) [28].

It is noteworthy that, although mTOR inhibitors have anti-neoplastic effects [29], their use was low in our study, with few patients adopting mTOR-based therapies following post-malignancy diagnosis. Prior studies demonstrated that the use of mTOR inhibitors reduced the occurrence of new malignancies and enhanced the prognosis for patients with pre-existing malignancies [30]. With growing evidence of their oncologic benefits, wider use of mTOR inhibitors after a malignancy diagnosis may be warranted. Conversion to mTORi can reduce or delay non-melanoma skin cancer occurrence but may be limited by discontinuation and adverse-event burden, underscoring the need for individualized selection [31].

Lower HLA compatibility showed a non-significant trend toward higher malignancy rates. Prior studies indicate a correlation between increased HLA mismatch and higher immunosuppression needs, potentially leading to elevated malignancy risk [32]. Yet, research results are inconsistent, requiring more data for a clearer understanding of this connection.

Survival differed significantly based on malignancy type; this was a major discovery. Survival outcomes were significantly poorer for individuals diagnosed with Kaposi sarcoma and PTLD, in contrast to those with other skin cancers or urinary tract cancers. Prior research aligns with this, indicating that systemic, hematological, and virus-linked malignancies predict poorer outcomes than localized solid tumors [33]. CNS involvement and multi-organ disease in PTLD patients usually predict a poor prognosis [34].

Treatment approaches differed considerably depending on the type of malignancy. Surgery was mainly used for skin and urinary tumors, but PTLD often needed CT or CT and RT. Though vital in managing PTLD, immunosuppression reduction was more frequent among those with Kaposi sarcoma. Lowering immunosuppression may induce remission in early Kaposi sarcoma and some PTLD, though this must be weighed against transplant rejection [10].

Although the pre-transplant malignancy rate was low (7%), post-transplant outcomes mirrored those observed in patients with de novo malignancies. This finding is reassuring and confirms the existing view that, following a suitable disease-free interval [35], patients with a history of treated malignancy may be considered for transplantation.

Univariate analysis revealed significant associations, but multivariate logistic regression modeling revealed no statistically significant independent predictors of mortality for renal transplant recipients with post-transplant malignancy. A trend towards increased mortality risk with older age at transplantation (OR = 1.026) was observed, but lacked statistical significance, supporting prior studies that age alone is an inadequate predictor of mortality without considering comorbidities and cancer aggressiveness [15]. Similarly, while EBV seropositivity is a known risk factor for PTLD death [24,26], our study did not find it to be an independent predictor, possibly due to confounding from concurrent risk variables or limited statistical power.

Interestingly, CNI maintenance had a protective association (OR = 0.132), suggesting potential immunomodulatory effects; however, the wide CI and *p*-value > 0.05 undermine definitive conclusions. Research has shown CNIs play a dual part in cancer, suppressing T cell immunity and potentially exhibiting direct anti-tumor activity [28]. Conversely, increased mortality risk was associated with antimetabolite use (OR = 3.207), although insignificantly, consistent with prior research showing varied links between azathioprine/mycophenolate mofetil and post-transplant cancer outcomes [29]. The combined results highlight the multifaceted nature of mortality in cancer patients post-kidney transplant, defying prediction from individual clinical or immunological markers. Future prospective studies with larger sample sizes and stratification by malignancy subtype are needed to delineate independent mortality predictors more clearly.

This study benefits from a large sample size and a long-term follow-up period, allowing for a detailed characterization of post-transplant malignancies and associated outcomes. The comprehensive dataset and multi-decade scope provide valuable insights into evolving clinical patterns. However, certain limitations should be acknowledged. First, its retrospective single-center design may introduce selection bias and limit the ability to infer causality. Second, underreporting of certain clinical outcomes is possible, particularly due to loss to follow-up over the long study period (1976–2019). Third, changes in clinical practice, diagnostic capabilities, and immunosuppressive protocols over more than four decades may have introduced variability that could influence outcomes. In addition, some variables—such as EBV and HHV-8 viral loads or detailed induction therapy records—were incomplete, limiting subgroup analyses. Finally, the heterogeneity of baseline immunosuppressive regimens and oncological diagnoses precluded the application of a standardized immunosuppression tapering protocol after malignancy diagnosis.

## 5. Conclusions

Our findings highlight the substantial burden of malignancies among renal transplant recipients, significantly compromising both patient and graft survival. Effective risk mitigation strategies should include individualized immunosuppression management, routine viral monitoring, and broader use of mTOR inhibitors with potential antineoplastic benefits. Given the high incidence and aggressive nature of post-transplant skin cancers, annual dermatological screening and early biopsy of suspicious lesions are critical. Ultimately, a personalized post-transplant surveillance model, emphasizing early detection and targeted intervention, is essential to improving long-term outcomes in this vulnerable population.

## Figures and Tables

**Table 1:** Demographic variables of transplant patients.

Variables	Total (*n* = 163), *n* (%)
Sex (F/M)	56 (34.4)/107 (65.5)
Follow-up Time (months) *	157 (7–531)
Duration of Renal Replacement Therapy (months) *	84 (13–156)
Age of TX *	40.0 (13.0–72.0)
Age of Malignancy Diagnosis *	50 (23.0–78.0)
Smoking status	28 (23.7)
eGFR at Diagnosis (mL/min) *	59.0 (8.0–117.0)
ESRD etiology	
Chronic GN	29 (17.8)
CAKUT	13 (8.0)
HT	10 (6.1)
PRD	10 (6.1)
Amyloidosis	8 (4.9)
VUR nephropathy	7 (4.2)
DM	5 (3.1)
Others	5 (3.1)
Unknown	76 (46.6)
Donor type	
Deceased	53 (32.5)
Living	110 (67.5)
HLA histocompatibility ***	
0	6 (4.9)
I	5 (4.1)
II	13 (10.7)
III	74 (60.7)
IV	11 (9.0)
V	5 (4.1)
VI	8 (4.8)
DM-Post-TX	42 (26.6)
History of acute rejection	13 (7.7)
Acute Rejection Period	
Within first year	12 (92.3)
After first year	1 (7.6)
Graft loss	20 (12.3)
Causes of Graft Loss	
Chronic Allograft Nephropathy	16 (9.3)
Chronic AMR	2 (1.3)
TCMR + AMR	1 (0.6)
AA Amyloidosis	1 (0.6)
EBV Serology	38 (23.3)
CMV Serology	82 (50.3)
HBV Serology	10 (6.1)
HCV Serology	35 (21.5)
BKV Serology	12 (7.4)
Pre-TX Dialysis Duration *	22.0 (1.0–185.0)
Pre-TX Dialysis	143 (87.7)
Pre-TX Dialysis Type	
HD	121 (76.5)
PD	12 (7.6)
PD + HD	6 (3.8)
Retransplantation	4 (2.5)
Unknown	5 (3.2)
Induction Regimen **	
ATG/ALG	47 (28.8)
Anti-IL-2	4 (2.5)
HLA histocompatibility ***	
Low	92 (79.3)
High	24 (20.7)
DM-Pre-TX	12 (7.3)
Maintenance treatment	
Steroids	161 (98.8)
CsA	113 (69.3)
MMF-MYF	80 (49.1)
AZA	78 (47.9)
FK	44 (27.0)
EVO	2 (1.2)
RAPA	2 (1.2)
Number of rejections	
I	11 (84.6)
II	2 (15.4)
Rejection treatment	
Pulse steroid	8 (61.5)
Pulse steroid + ATG	4 (30.8)
IVIG	1 (7.7)
Death-censored graft survival	
Actual Graft Loss	20 (12.0)
Death-Censored	51 (30.5)
Mortality	93 (57.1)

Notes: * Numeric variables were presented as median (minimum–maximum) or mean ± SD. ** Induction Regimen information was not available for 108 patients. *** HLA matching was assessed in *n* = 47 patients with available data; percentages were calculated using this denominator. Seropositivity indicates at least one documented positive serology during follow-up. Abbreviations: ALG/ATG: Antithymocyte globulin/Antilymphocyte globulin, AMR: Antibody-Mediated Rejection, AZA: Azathiopurine, BKV: BK virus, CAKUT: Congenital abnormalities of the kidney and urinary tracts, CMV: Cytomegalovirus, CsA: Cyclosporine A, DM: Diabetes Mellitus, EBV: Epstein–Barr virus, eGFR: estimated Glomerular Filtration Rate, ESRD: End-stage Renal Disease, EVO: Everolimus, FK: Tacrolimus, GN: glomerulonephritis, HBV: hepatitis B, HCV: hepatitis C, HD: hemodialysis, HLA: Human Leucocyte Antigen, IL: Interleukin, IVIG: Intravenous Immunoglobulin, min: minute, mL: milliliter, MMF: Mycophenolate mofetil, MYF: Mycophenolate Sodium, PRD: Polycystic Renal Diseases, PD: Peritone Dialysis, RAPA: Rapamycin, TCMR: T Cell-Mediated Rejection, TX: Transplantation, VUR: Vesicoureteral reflux.

**Table 2:** Characteristics of malignancy status of transplant patients.

Variables	Total (*n* = 163), *n* (%)
Presence of Pre-TX Malignancy *	11 (6.8)
Type of Pre-TX malignancy	
Non-melanoma Skin Tumor	1 (9.1)
Breast	2 (18.2)
Colon	1 (9.1)
RCC	1 (9.1)
Bladder	1 (9.1)
Intracranial	2 (18.2)
Others	3 (27.3)
Organ Dysfunction at diagnosis in those with PTLD	9 (52.9)
Post-Diagnosis IS Regimen	
Same	29 (17.7)
Combination with mTOR	97 (59.5)
Combination Without mTOR	34 (20.9)
Post-TX malignancy *	160 (95.8)
Type of Post-TX malignancy	
Non-Kaposi Sarcoma Skin Tumor	48 (30.0)
Kaposi Sarcoma	18 (11.1)
PTLD	17 (10.5)
Urinary	13 (8.0)
Other Solid Tumor	52 (31.9)
Other Hematological Tumor	6 (3.7)
Premalignant lesions	6 (3.7)
CNS involvement in patients with PTLD	4 (23.5)
Treatment for Malignancy	
Surgery + CT/RT	30 (18.4)
Surgery	78 (47.8)
CT	12 (7.4)
CT + RT	5 (3.1)
Reduction of immunosuppression	14 (8.6)
Surgery + RAI	4 (2.4)
RT	2 (1.5)
Follow-up without treatment	18 (11.0)

Notes: * 11 patients (6.7%) had a pre-transplant malignancy, and 160 patients (98.2%) had a post-transplant malignancy. Three patients (1.8%) were diagnosed with both a pre- and a post-transplant malignancy. Abbreviations: CT: Chemotherapy, IS: Immunosuppressive, mTOR: Mammalian Target of Rapamycin, PTLD: Post-transplant lymphoproliferative disorder, RAI: Radioactive iodine, RCC: Renal Cell Carcinoma, RT: Radiotherapy, TX: Transplantation.

**Table 3:** Characteristics of age, sex, induction regimen, and acute rejection history according to malignancy type.

	Kaposi Sarcoma *n* = 18, *n* (%)	Non-Kaposi Sarcoma Skin Tumor *n* = 48, *n* (%)	PTLD *n* = 17, *n* (%)	Urinary *n* = 13, *n* (%)	Other Solid Tumor *n* = 52, *n* (%)	Other Hematological Tumor *n* = 6, *n* (%)	Premalignant Lesions *n* = 6, *n* (%)
Age *	59.3 ± 12.8	62.0 ± 8.8	55.6 ± 10.2	66.2 ± 12.5	56.2 ± 12.0	54.2 ± 13.7	47.3 ± 7.7
Sex (F/M)	3 (16.7)/15 (83.3)	9 (18.8)/39 (81.3)	6 (35.3)/11 (64.7)	2 (15.4)/11 (84.6)	25 (48.1)/27 (51.9)	2 (33.3)/4 (66.7)	5 (83.3)/1 (16.7)
Induction Regimen **							
ATG/ALG	3 (16.7)	8 (16.7)	9 (52.9)	3 (23.1)	8 (34.6)	2 (33.3)	1 (16.7)
Anti-IL-2	0 (0.0)	0 (0.0)	0 (0.0)	0 (0.0)	1 (1.9)	0 (0.0)	1 (16.7)
History of acute rejection	3 (16.7)	4 (8.3)	1 (5.9)	0 (0.0)	5 (9.6)	0 (0.0)	1 (16.7)

Notes: * Numeric variables were presented as mean ± SD. ** Induction Regimen information was not available for 108 patients. Abbreviations: ALG/ATG: Antithymocyte globulin/Antilymphocyte globulin, IL: Interleukin.

**Table 4:** Characteristics of participants according to mortality status.

	Survivors, *n* = 70, *n* (%)	Non-Survivors, *n* = 93, *n* (%)	*p*-Value
Age of Malignancy Diagnosis	45.6 ± 10.5	52.6 ± 12.1	<0.001
Sex (F/M)	34 (48.6)/36 (51.4)	22 (23.7)/71 (76.4)	0.001
Follow-up Time (months)	162.5 (15/561)	150 (7–473)	0.299
Age of TX	37.3 ± 10.8	43.7 ± 12.2	<0.001
Duration of Renal Replacement Therapy (months)	84 (13–156)	36 (13–36)	0.018
Smoking status	11 (22.9)	17 (24.3)	>0.999
Donor type			0.930
Deceased	22 (31.4)	31 (33.3)	
Living	48 (68.6)	62 (66.7)	
HLA histocompatibility			0.030
0	5 (8.8)	1 (1.5)	
I	3 (5.3)	2 (3.2)	
II	2 (3.5)	11 (16.3)	
III	34 (59.7)	40 (61.5)	
IV	8 (14.0)	3 (4.6)	
V	3 (5.3)	2 (3.1)	
VI	2 (3.5)	6 (9.2)	
HLA histocompatibility			0.422
Low	39 (75.0)	53 (82.8)	
High	13 (25.0)	11 (17.2)	
EBV Serology	23 (32.9)	15 (16.1)	0.026
CMV Serology	39 (55.7)	43 (46.2)	0.460
HBV Serology	3 (4.3)	7 (7.5)	0.285
HCV Serology	12 (17.1)	23 (24.7)	0.076
BKV Serology	8 (11.4)	4 (4.3)	0.092
ATG/ALG	20 (28.6)	27 (29.0)	0.281
Anti-IL2	3 (4.3)	1 (1.2)	0.161
CNI drugs			0.017
FK	27 (38.6)	17 (18.3)	
CsA	41 (58.6)	72 (77.4)	
Antimetabolite drugs			0.006
MMF-MYF	43 (61.4)	37 (39.8)	
AZA	27 (38.6)	51 (54.8)	
mTOR drugs			>0.999
EVO	1 (1.4)	1 (1.1)	
RAPA	1 (1.4)	1 (1.1)	
Steroid	70 (100.0)	91 (97.9)	0.507
History of acute rejection	7 (10.0)	6 (6.5)	0.592
Within first year acute rejection	7 (100.0)	5 (83.3)	0.462
Number of rejections			0.462
I	5 (71.4)	6 (100.0)	
II	2 (28.6)	0 (0.0)	
Rejection treatment			0.414
Pulse steroid	5 (71.4)	3 (50.0)	
Pulse steroid + ATG	1 (14.3)	3 (50.0)	
IVIG	1 (14.3)	0 (0.0)	
Presence of Pre-TX Malignancy	7 (10.0)	4 (4.3)	0.209
Type of Pre-TX malignancy			0.050
Non-melanoma Skin Tumor	1 (14.3)	0 (0.0)	
Breast	0 (0.0)	2 (50.0)	
Colon	0 (0.0)	1 (25.0)	
RCC	0 (0.0)	1 (25.0)	
Bladder	1 (14.3)	0 (0.0)	
Intracranial	2 (28.6)	0 (0.0)	
Others	3 (42.9)	0 (0.0)	
Graft loss	7 (10.0)	10 (10.8)	>0.999
Type of Post-TX malignancy			0.005
Non-Kaposi Sarcoma Skin Tumor	7 (11.1)	9 (9.9)	
Kaposi Sarcoma	16 (25.4)	30 (33.0)	
PTLD	6 (9.5)	11 (12.1)	
Urinary	2 (3.2)	10 (11.0)	
Other Solid Tumor	24 (38.1)	27 (29.7)	
Other Hematological Tumor	4 (6.4)	2 (2.2)	
Premalignant lesions	4 (6.4)	2 (2.2)	

Abbreviations: ATG/ALG: Antithymocyte globulin/Antilymphocyte globulin, AZA: Azathiopurine, BKV: BK virus, CMV: Cytomegalovirus, CNI: Calcineurin inhibitors, CsA: Cyclosporine A, DM: Diabetes Mellitus, EBV: Epstein–Barr virus, EVO: Everolimus, FK: Tacrolimus, HBV: hepatitis B, HCV: hepatitis C, HLA: Human Leucocyte Antigen, IL: Interleukin, IVIG: Intravenous Immunoglobulin, MMF: Mycophenolate mofetil, MYF: Mycophenolate Sodium, PTLD: Post-transplant lymphoproliferative disorder, RAPA: Rapamycin, RCC: Renal Cell Carcinoma, TX: Transplantation.

**Table 5:** Evaluation of pre- and post-transplant cancer groups according to HLA compatibility status.

	Low HLA Histocompatibility, *n* = 94, *n* (%)	High HLA Histocompatibility, *n* = 24, *n* (%)	*p*-Value
Type of Pre-TX malignancy			>0.999
Non-melanoma Skin Tumor	1 (12.5)	0 (0.0)	
Breast	1 (12.5)	0 (0.0)	
Colon	1 (12.5)	0 (0.0)	
RCC	0 (0.0)	0 (0.0)	
Bladder	1 (12.5)	0 (0.0)	
Intracranial	2 (25.0)	0 (0.0)	
Others	2 (25.0)	0 (0.0)	
Type of Post-TX malignancy			0.408
Non-Kaposi Sarcoma Skin Tumor	12 (13.5)	3 (12.5)	
Kaposi Sarcoma	27 (30.3)	7 (29.2)	
PTLD	10 (11.2)	2 (8.3)	
Urinary	3 (3.4)	3 (12.5)	
Other Solid Tumor	31 (34.8)	7 (29.2)	
Other Hematological Tumor	2 (2.3)	2 (8.3)	
Premalignant lesions	4 (4.5)	0 (0.0)	

Abbreviations: PTLD: Post-transplant lymphoproliferative disorder, TX: Transplantation.

**Table 6:** Multivariate logistic regression analysis of factors associated with mortality.

	*p*-Value	OR	95% CI	
			Lower	Upper
Age at Transplantation	0.678	1.026	0.910	1.156
EBV Serostatus	0.383	0.260	0.013	5.356
Maintenance Therapy (Calcineurin Inhibitor)	0.111	0.132	0.011	1.591
Maintenance Therapy (Antimetabolite)	0.404	3.207	0.208	49.434

Abbreviations: CI: Confidence Interval, EBV: Epstein–Barr virus, OR: Odds Ratio.

## Data Availability

The data that support the findings of this study are available from the corresponding author upon reasonable request.

## Supplementary Materials

The following supporting information can be downloaded at https://www.mdpi.com/article/10.3390/jcm14165858/s1, Figure S1: Study cohort selection and follow-up flow chart.
