# Supplementary figures and images for: Malignancies After Renal Transplantation: Frequency, Etiology, and Prognosis—A Single Center Experience
Source: J Clin Med. 2025 Aug 19;14(16):5858. doi: 10.3390/jcm14165858 (PMC12387312; doi:10.3390/jcm14165858)

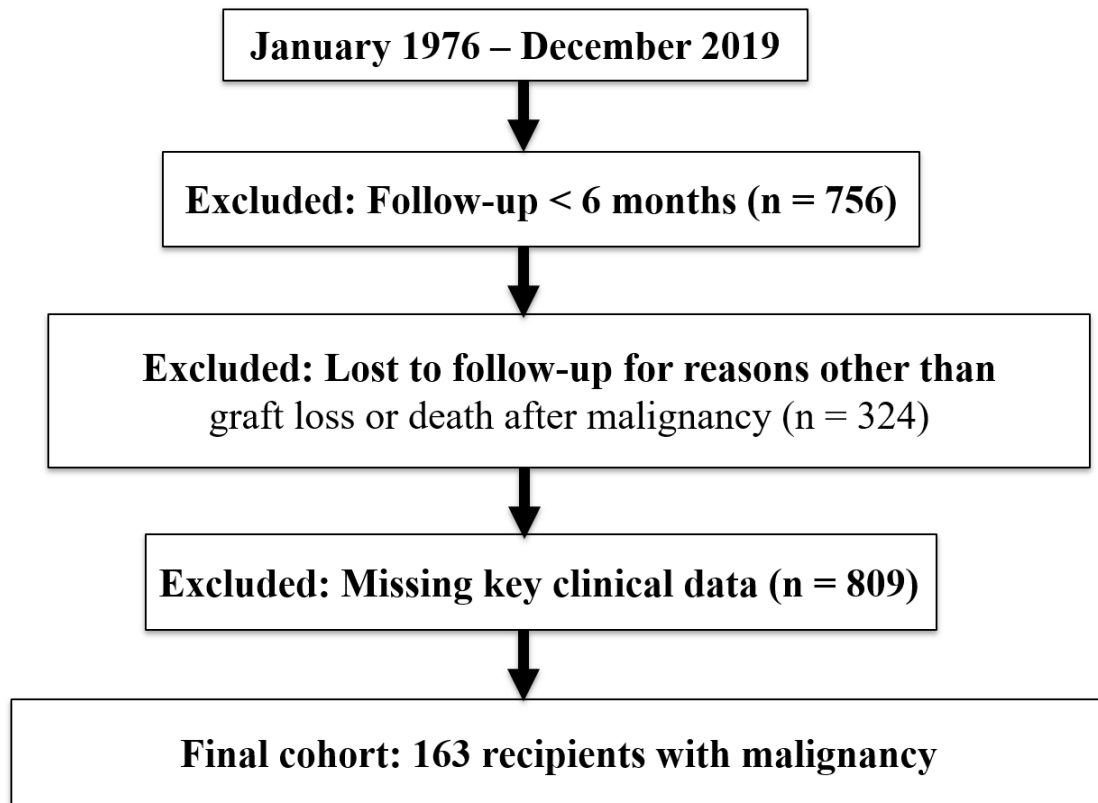

**Supplemental Figure 1.** Study cohort selection and follow-up flow chart

Supplement: Supplementary file 1 [file jcm-14-05858-s001.zip › jcm-3792055-supplementary.pdf]
